# Apremilast, a novel PDE4 inhibitor, inhibits spontaneous production of tumour necrosis factor-alpha from human rheumatoid synovial cells and ameliorates experimental arthritis

**DOI:** 10.1186/ar3041

**Published:** 2010-06-02

**Authors:** Fiona E McCann, Andrew C Palfreeman, Melanie Andrews, Dany P Perocheau, Julia J Inglis, Peter Schafer, Marc Feldmann, Richard O Williams, Fionula M Brennan

**Affiliations:** 1 The Kennedy Institute of Rheumatology, Imperial College London, 65 Aspenlea Road, London, W6 8LH, UK; 2 Institute for Molecular Bioscience, University of Queensland, Bldg 80 Services Road, Brisbane, QLD 4072, Australia; 3 Pharmacology & Anaesthesiology Unit, School of Medicine & Pharmacology, University of Western Australia, 35 Stirling Highway, Crawley, WA 6009, Australia; 4 Translational Development, Celgene Corporation, 86 Morris Avenue, Summit, NJ, 07901, USA

## Abstract

**Introduction:**

Type 4 phosphodiesterases (PDE4) play an important role in immune cells through the hydrolysis of the second messenger, cAMP. Inhibition of PDE4 has previously been shown to suppress immune and inflammatory responses, demonstrating PDE4 to be a valid therapeutic target for immune-mediated pathologies. We assessed the anti-inflammatory effects of a novel PDE4 inhibitor, apremilast, in human synovial cells from rheumatoid arthritis (RA) patients, as well as two murine models of arthritis.

**Methods:**

Cells liberated from tissue excised from arthritic joints of RA patients were cultured in the presence of increasing concentrations of apremilast for 48 hours and spontaneous tumour necrosis factor-alpha (TNFα) production was analysed in culture supernatants by ELISA. In addition, arthritis was induced in BALB/c and DBA/1 mice by passive transfer of anti-type II collagen mAb and immunisation with type II collagen, respectively. Mice with established arthritis received 5 or 25 mg/kg apremilast and disease severity was monitored relative to mice receiving vehicle alone. At the end of the study, paws were removed and processed for histopathological assessment. Behavioural effects of apremilast, relative to rolipram, were assessed in naïve DBA/1 mice using an automated activity monitor (LABORAS).

**Results:**

Apremilast dose dependently inhibited spontaneous release of TNFα from human rheumatoid synovial membrane cultures. Furthermore, apremilast significantly reduced clinical score in both murine models of arthritis over a ten day treatment period and maintained a healthy joint architecture in a dose-dependent manner. Importantly, unlike rolipram, apremilast demonstrated no adverse behavioural effects in naïve mice.

**Conclusions:**

Apremilast is an orally available PDE4 inhibitor that reduces TNFα production from human synovial cells and significantly suppresses experimental arthritis. Apremilast appears to be a potential new agent for the treatment of rheumatoid arthritis.

## Introduction

There has been significant progress in the treatment of rheumatoid arthritis (RA), particularly with the development of anti-TNFα therapy. The anti- TNFα biologicals currently in use (infliximab, etanercept and adalimumab) are highly effective in reducing inflammation and limiting joint destruction [1,2]. However, this costly treatment is administered via repeated injections; hence, there is a need for cheaper, orally available treatments that reduce the production of TNFα and other inflammatory mediators. A much explored pharmacological method to inhibit TNFα production is via the inhibition of Type 4 phosphodiesterases (PDE4). PDE4 inhibitors are synthetic, small molecular weight compounds that are orally available and have been demonstrated to reduce TNFα production in human and mouse lymphocytes and macrophages [3,4].

There are 11 families in the PDE group, many of which contain a number of subtypes [5]. PDE4 is a cyclic adenosine monophosphate (cAMP) specific enzyme, which hydrolyses cAMP to AMP and is the predominant isoenzyme expressed in macrophages, lymphocytes and neutrophils [6]. Elevation of intracellular cAMP, via inhibition of PDE4, triggers the protein kinase A pathway, inhibits TNFα production and suppresses the immune response [7-9]. Although the anti-inflammatory properties of PDE4 inhibitors could be exploited for the treatment of an array of inflammatory diseases, no PDE4 inhibitors have been approved for clinical use due to problems with toxicity [10]. PDE4 was initially chosen as a target in the treatment of airway inflammation due to its expression in the airways [11,12]. At present, roflumilast [13] is pending regulatory approval for the treatment of chronic obstructive pulmonary disease (COPD) [14].

Although there are a number of PDE4 inhibitors currently available to researchers, most induce side effects of nausea and emesis. Other reported side effects include headaches, diarrhoea, heart failure and arrhythmias [15,16]. A novel PDE4 inhibitor, apremilast has recently been generated [17] which has a half maximal inhibitory concentration (IC_50_) of 74 nM and inhibits TNFα production from lipopolysaccharide (LPS)-stimulated human peripheral blood mononuclear cells (PBMC) and whole blood by 7.7 nM and 11 nM, respectively [17]. Most recently, apremilast has exhibited broad anti-inflammatory effects *in vitro*, through the inhibition of multiple mediators, including TNFα, interferon (IFN)γ, granulocyte macrophage-colony stimulating factor, IL-12 and IL-23 in LPS-stimulated human monocytes, with similar effects on TNFα reported in human NK cells and keratinocytes, two cell types involved in psoriasis pathophysiology [18]. Furthermore, during the course of our studies, apremilast has entered phase II clinical trials for the treatment of psoriasis, psoriatic arthritis (PsA), and other inflammatory diseases. Out of 168 patients with PsA participating in a phase II randomized, double-blind, placebo controlled, study conducted in North America and Europe, 44% met the primary endpoint of ACR20 (improvement of symptoms by 20% according to American College of Rheumatology score) after 12 weeks on 20 mg apremilast twice daily compared with 12% of the placebo group [19]. In addition, the effects of apremilast have been tested on a small group of patients with severe plaque-type psoriasis [20]. Fourteen of seventeen patients demonstrated an improvement in Psoriasis Area and Severity Index scores. Apremilast has also been reported to down-regulate intracellular IL-6 in cell lysates of myeloma cell and human umbilical vein endothelial cells co cultures [21]. As TNFα blockade is known to be an exceedingly effective therapeutic approach in many patients with ankylosing spondylitis, the effects of apremilast in ankylosing spondylitis are currently being tested in a phase II, randomised, double-blinded, clinical control study at our centre.

Here, we demonstrate that apremilast inhibits spontaneous production of TNFα, but not IL-6 or IL-10 from *ex-vivo *cultures of human rheumatoid synovial membranes. Thus, to determine the anti-arthritic capacity of apremilast, we treated mice with two different forms of established experimental arthritis. Disease severity was evaluated throughout, followed by histological assessment of the extent of joint inflammation and erosion at the end of the treatment period. Our findings show that apremilast has potent disease-modifying properties, but, crucially, lacks the behavioural effects exhibited by the classical PDE4 inhibitor, rolipram.

## Materials and methods

### LPS-stimulated monocytes

Buffy coats were purchased from the North London Blood Bank and cells were separated over a density gradient to obtain a population of PBMCs. The PBMCs were further separated into lymphocyte, monocyte and granulocyte populations by centrifugal elutriation. Monocytes were plated out in triplicate into a 96-well flat bottom plate at 1 × 10^5 ^cells/well in RPMI containing 5% heat-inactivated FCS. The monocytes were then treated with increasing concentrations of apremilast (Celgene Corporation, Summit, New Jersey, USA), rolipram (Sigma Aldrich, Dorset, UK) and a vehicle control consisting of 3.3 × 10^-4 ^% dimethyl sulphoxide (DMSO), the diluent for the highest concentration of drug, for 30 minutes. The pre-treated cells were then stimulated with 10 ng/ml LPS and cultured for 24 hours at 37°C and 5% carbon dioxide. Cytokines in cell culture supernatants was determined by ELISA (BD Pharmingen, San Diego, CA, USA), following the manufacturers' instructions. Absorbance was read and analysed at 450 nm on a spectrophotometric ELISA plate reader (Labsystems Multiskan Biochromic, Vienna, VA, USA) using the Ascent version 2.4.2 software (ThermoFisher Scientific, Waltham, MA, USA).

### Human rheumatoid synovial membrane cell cultures

Human rheumatoid synovial membrane samples were obtained from RA patients undergoing joint replacement surgery, following informed consent and anonymisation. Experiments were performed at KIR, Imperial College London and approval was obtained from the Riverside Research Ethics Committee, UK. The samples were teased apart and dissected into tiny pieces before enzymatic digestion with DNase and collagenase (type IV), as previously described [22,23]. Once isolated, the cells were plated in a flat-bottomed 96-well plate in triplicate at 1 × 10^6 ^cells/well in RPMI containing 10% heat-inactivated FCS. Cells were then treated with increasing concentrations of apremilast, rolipram or a vehicle control consisting of 3.3 × 10^-4 ^% DMSO. As a positive control, cells were treated with a combination of anti-TNFα mAb and IL-1RA (both at 10 μg/ml; R&D Systems, Greater Minneapolis, MN, USA). The treated cells were cultured for 48 hours at 37°C and 5% carbon dioxide, before supernatants were harvested and analysed by ELISA, as described above. Cell viability was assessed by assaying uptake and metabolism of 3-(4,5-Dimethylthiazol-2-yl)-2,5-diphenyltetrazolium bromide (MTT) as previously described [24].

### Induction and assessment of arthritis - mAb-induced arthritis

mAb-induced arthritis experiments were carried out by MD Biosciences/Harlan, Israel. According to the protocol described by Terato and colleagues [25], collagen mAb-induced arthritis was achieved by initial injection of a four-component arthritogenic mAb cocktail and the subsequent administration of LPS. Monoclonal antibodies were D1, F10, A2 and D8 clones raised against CB11, a CNBr-generated arthritogenic fragment of chick type II collagen. On day 0, six-week-old male BALB/c mice were given a single intravenous injection of mAb cocktail at a dose of 100 mg/kg (about 2 mg/mouse). Three days later, mice received an intraperitoneal injection of 2.5 mg/kg LPS (about 50 μg/mouse). One hour prior to administration of LPS, apremilast or vehicle alone (0.5% carboxymethyl cellulose, 0.25% Tween 80) was administered by oral gavage, and then daily for four days until day 7. Arthritis was monitored in each animal by measuring paw thickness with micro-calipers and by applying the following clinical scoring system according to the in-house scale of Morwell MD Biosciences Inc. (Nes-Ziona, Israel): 0, normal; 1, mild swelling and redness restricted to digits; 2, moderate swelling and redness of ankle; 3, severe redness and swelling of the entire paw including digits; 4, maximally inflamed limb with involvement of multiple joints, until day 9 when mice were sacrificed. Hind paws, including ankle joints, were removed and processed for paraffin wax embedding and histology. This study was approved by the Committee for Ethical Conduct in the Care and use of Laboratory animals of the Hebrew University, Jerusalem.

### Induction and assessment of arthritis - collagen-induced arthritis

Collagen-induced arthritis (CIA) experiments were performed at KIR, Imperial College London. Ten-week-old male DBA/1 mice were immunised by intradermal injection at the base of the tail with 200 μg bovine type II collagen (2 mg/ml) in complete Freund's adjuvant (CFA) [26]. When mice began to develop signs of arthritis, treatment with apremilast or vehicle (0.5% carboxymethyl cellulose, 0.25% Tween 80) was initiated. Mice were treated daily by intraperitoneal (i.p.) injection for 10 days post onset, and disease severity was monitored throughout, according to the following score: 0, normal, 1, slight swelling and/or erythema, 2, pronounced oedematous swelling. At the end of the study, paws were removed and processed for histological assessment. All procedures were approved by the Ethical Review Process Committee and the UK Home Office.

### Histopathological assessment of joints

Joints were fixed in 10% neutral buffered formalin for one week, then transferred into buffered inorganic acid for decalcification for 48 hours, and then back into 10% formalin prior to storage. Joints were trimmed and embedded in paraffin and sections of 6 μm were cut and stained in H&E. Histopathological changes in the joints from mAb-induced arthritis were described and scored, using semi-quantitative grading of five scores (0, unremarkable, 1, minimal, 2, mild, 3, moderate, 4, marked). CIA was scored as follows: 0, normal; 1, minimal synovitis without cartilage/bone erosion; 2, synovitis with some marginal erosion but joint architecture maintained; 3, severe synovitis and erosion with loss of normal joint architecture.

### Assessment of spontaneous behaviour using LABORAS

The Laboratory Animal Behaviour Observation, Registration and Analysis System, (LABORAS, Metris B.V., Hoofddorp, The Netherlands), is an automated system that detects vibrations evoked by movement of a single rodent in a cage. Pattern recognition software is used to recognise and quantify different behaviours, including grooming, mobility, climbing and immobility [27]. Spontaneous behaviour of naive mice treated with apremilast (25 mg/kg i.p), rolipram (25 mg/kg i.p.) or vehicle alone was assessed for 30 minutes, one hour after dosing, using LABORAS as described [28]. The time spent engaging in specified activities or immobility was measured. Animals were acclimatised to the equipment on two occasions prior to measurement.

### Lymph node cultures

Inguinal lymph nodes from bCII immunised DBA/1 mice were excised at day 14 post immunisation and cells were dissociated and plated out in a U bottom 96-well plate at 2 × 10^6 ^cells/ml in RPMI 1640 containing 10% fetal bovine serum, 50 U/ml penicillin/streptomycin and 50 μM 2-mercaptoethanol. Cells were stimulated with 100 ng/ml anti-CD3 mAb (145-2C11) or 50 μg/ml bCII, or left unstimulated. Cells were cultured for 48 hours before 100 μl of supernatant was removed for subsequent cytokine analysis by ELISA and the remainder was pulsed with 1 μCi per well with ^3^H-thymidine to measure proliferation. Pulsed cells were cultured for a further 20 hours before thymidine incorporation was measured.

### Statistical analysis

Two-way analysis of variance (ANOVA), followed by Bonferroni multiple comparison test was employed for analysis of paw thickness on days post onset of arthritis, spontaneous behaviour in LABORAS and *in vitro *cytokine production and proliferation of lymph node cultures. All other data was analysed by one-way ANOVA followed by Dunnett's multiple comparison test. Calculations were made using GraphPad Prism software.

MAb-induced arthritis experiments were carried out at MD Biosciences (Harlan, Israel). All other experiments were performed at KIR, Imperial College London.

## Results and discussion

To date, several PDE4 inhibitors have been tested in experimental arthritis due to their potent capacity to inhibit TNFα production in a range of cell types [3,4,12,29,30]. Previously, our group and others have demonstrated the anti-arthritic effects of PDE4 inhibitors in experimental arthritis [31-34]. More recently, Yamamoto and colleagues [35] has reported that a dual PDE7/PDE4 inhibitor, YM-393059, effectively ameliorates CIA. During the course of our studies, significant progress has been made with the second-generation PDE4 inhibitor roflumilast, now proving effective in clinical trials for asthma and COPD. Presently, there is no clinical data available for PDE4 inhibitors in the treatment of RA. Here, having established that the novel PDE4 inhibitor apremilast dose dependently inhibits TNFα release from LPS-stimulated monocytes and rheumatoid synovial membrane cultures, we next evaluated its anti-arthritic potential in two independently executed, acute mouse models of arthritis, namely mAb-induced arthritis and CIA. Both models have been extensively used in pre-clinical trials, demonstrating high degrees of similarity, in terms of cellular and humoral-mediated immunity to RA [36].

### Apremilast inhibits TNFα production by LPS-stimulated monocytes, and the spontaneous release of TNFα from human rheumatoid synovial membrane cultures

**Figure 1:**
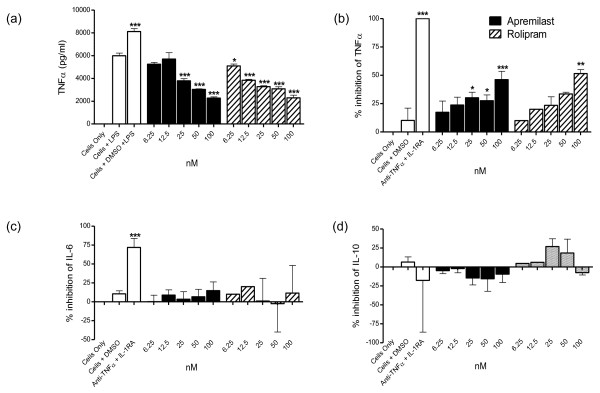
**Apremilast inhibits TNFα release from LPS-stimulated monocytes and human rheumatoid synovial membrane cultures**. Human peripheral blood monocytes were treated with increasing concentrations of apremilast, 30 minutes before stimulating with 10 ng/ml lipopolysaccharide (LPS) for 24 hours. **(a) **Culture supernatants were assayed for TNFα by ELISA. Data is representative of three donors. Human rheumatoid arthritis (RA) synovial membrane cells were cultured for 48 hours in the presence of apremilast, rolipram or controls and spontaneous production of **(b) **TNFα, **(c) **IL-6 and **(d) **IL-10 in culture supernatants was measured by ELISA. Means and standard errors of percent inhibition, from five (apremilast) or two (rolipram) donors are plotted. As a positive control, cells were treated with a combination of anti-TNFα mAb and IL-1RA. Statistical analysis was calculated by one-way analysis of variance and Dunnett's multiple comparison test. Each test group was compared to (a) *cells + LPS*, or (b to d) *cells only*. * *P *< 0.05, ** *P *< 0.01, *** *P *< 0.001.

It has been previously shown that TNFα production from LPS-stimulated human monocytes can be inhibited by PDE4 inhibitors [37]. We demonstrate that apremilast and rolipram inhibit TNFα production from LPS-stimulated human monocytes that have been cultured for 24 hours, in a dose-dependent manner with IC_50_s of 55 nM and 40 nM, respectively (Figure 1a). Data are representative of three experiments from different donors, all of which displayed a similar dose-dependent inhibition of TNFα by apremilast and rolipram. Addition of DMSO alone (vehicle control) prior to LPS stimulation of human monocytes increased TNFα production in this experiment; however, this was not consistently observed in all donors. Human rheumatoid synovial membrane cells were cultured for 48 hours and spontaneously produced 255.4 ± 37.5 pg/ml, 454.32 ± 81.6 ng/ml and 56.52 ± 9.9 pg/ml of TNFα, IL-6 and IL-10, respectively (mean ± standard error of the mean). As for the LPS-stimulated monocytes, these cultures were treated with concentrations of apremilast and rolipram ranging from 6.25 nM to 100 nM. As a positive control, cells were treated with a combination of anti-TNFα mAb and IL-1RA (both at 10 μg/ml; R&D Systems, Greater Minneapolis, MN, USA). Apremilast inhibited spontaneous TNFα production in a dose-dependent manner but did not achieve 50% inhibition, with a maximal inhibition of 46% at the highest dose of 100 nM being reached. Rolipram also inhibited TNFα release in a dose-dependent manner and achieved an inhibition of 52% at a dose of 100 nM (Figure 1b). Both IL-6 (Figure 1c) and IL-10 (Figure 1d) was unaffected by apremilast and rolipram. The effect of apremilast on IL-1β production in RA synovial membranes was not assayed here; however, Schafer and colleagues have recently reported that apremilast does not inhibit IL-1β production in LPS-stimulated human PBMC at concentrations up to 10 μM [18]. The inhibition of spontaneous TNFα production in human rheumatoid synovial membrane cultures by apremilast and rolipram was not due to cell death. MTT assays were performed on all cell cultures after the supernatants had been collected and neither inhibitor had any effect on cell viability [See Supplementary figure S1 in Additional file 1]. In accordance with our findings, several other PDE4 inhibitors have been reported to have minimal effects on IL-6 in human PBMC, while potently inhibiting both TNFα and IL-1β production [35,38,39]. Similarly, elevation of intracellular cAMP by the addition of up to 1 mM dibutyryl cAMP to RA synovial membrane cultures had no effect on spontaneous production of IL-10 [40].

### Apremilast reduces severity of mAb-induced arthritis in BALB/c mice

**Figure 2:**
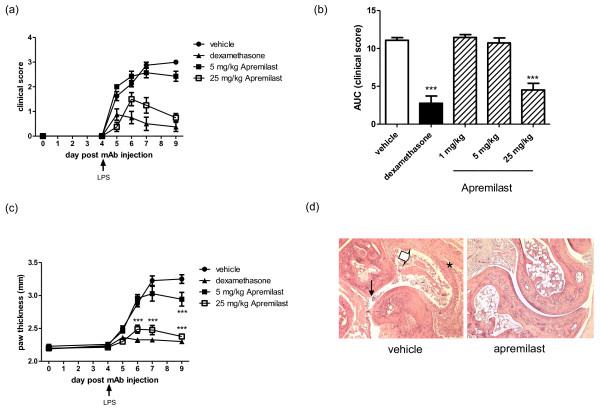
**Apremilast reduces severity of mAb-induced arthritis in BALB/c mice**. **(a) **Arthritic mice were treated orally with apremilast, dexamethasone or vehicle and disease severity was evaluated and assigned a clinical score. **(b) **Area under the curve for each mouse was calculated and statistical significance relative to vehicle control group was analysed (*** *P *< 0.001). **(c) **Paw thickness was measured throughout with microcalipers and statistical significance was calculated by two-way analysis of variance with Bonferroni post test analysis. Means and standard error of the mean are plotted, eight mice per group (*** *P *< 0.001). H&E staining of a longitudinal section through the tibio-tarsal joint from a vehicle-treated mouse and one treated with 25 mg/kg apremilast is shown in **(d)**. Asterisk shows inflammation of the synovial membrane, small arrow, erosion of articular cartilage, and bold arrow, fibrin deposits and inflammatory cell infiltrate within the articular cavity. Images were acquired at 100 × magnification.

Arthritis was induced in six-week-old male BALB/c mice by intravenous administration of a cocktail of four anti-collagen antibodies, followed by LPS (i.p) three days later. At this time, mice (eight per treatment) were given a daily, oral dose of vehicle or dexamethasone, to form negative and positive control groups, respectively, while experimental groups were treated orally (oral gavage) with 1, 5 or 25 mg/kg apremilast. Treatment continued for four successive days, until day 7, with close monitoring of disease severity throughout until day 9 (Figure 2). Two days after LPS administration (day 5 post injection of mAbs), all mice began to show varying degrees of arthritis severity. Apremilast at 5 and 25 mg/kg and dexamethasone at 1 mg/kg, significantly suppressed arthritis severity, as measured by clinical score (Figures 2a and 2b) and/or paw thickness (Figure 2c). Statistical significance of the clinical score of individual mice in all treatment groups relative to vehicle control, over the course of the treatment period, was assessed by measuring area under the curve per mouse, followed by one-way ANOVA and then Dunnett's multiple comparison test (Figure 2b). Similar to dexamethasone, the 25 mg/kg apremilast treatment group reached statistical significance (*P *< 0.001). Doses of 1 and 5 mg/kg did not reduce clinical score. On day 6, 7 and 9 post onset, hind paw thickness of mice treated with 25 mg/kg apremilast were significantly less than those receiving vehicle. It is also notable that on day 9 post onset, at the end of the treatment period, the 5 mg/kg treatment group also exhibited significantly less paw thickness relative to vehicle-treated mice (*P *< 0.001). Apremilast 1 mg/kg had no effect on paw swelling (data not shown), and as expected, dexamethasone completely abolished paw swelling throughout.

At the end of the experiment, hind paws including the ankle of each animal were removed and the tibio-tarsal joint was trimmed longitudinally, before processing for H&E staining to visualise the extent of inflammation and joint damage. Histopathological changes in the joints were described and scored, as detailed in materials and methods. Scores from the parameters assessed are described in Table 1. Although the majority of vehicle-treated mice scored mild or moderate for most parameters, such as pannus formation and cartilage disruption, apremilast-treated joints were evaluated as unremarkable or normal throughout. Figure 2d shows a representative longitudinal section through the tibio-tarsal joint of a vehicle and apremilast (25 mg/kg) treated mouse. Moderate inflammation of the synovial membrane (asterisk) is observed in the section from the vehicle-treated mouse (Figure 2d). Erosion of articular cartilage (small arrow) as well as fibrin deposits and inflammatory cell infiltrate within the articular cavity (bold arrow) is also evident. In contrast, there is no apparent inflammation of the tibio-tarsal joint from the mouse treated with 25 mg/kg apremilast, and the joint architecture has remained intact, similar to that of a healthy, non-arthritic mouse.

**Table 1:** Histopathological features observed in the tibio-tarsal joint of arthritic mice treated with vehicle or 25 mg/kg apremilast

	Vehicle								Apremilast							
		
Animal #	1	2	3	4	5	6	7	8	1	2	3	4	5	6	7	8
Synovial hyperplasia	1	2	1	2	2	1	1	1	0	0	0	0	0	0	0	0
Synovial villus formation	1	2	1	2	2	1	1	1	0	0	0	0	0	0	0	0
Fibrin deposition	2	3	3	3	3	2	3	3	0	0	0	0	0	1	0	1
Inflammatory infiltrate in the synovial membrane	3	3	3	3	3	3	3	3	0	0	0	0	0	0	0	0
Pannus formation	3	3	3	3	3	3	3	3	0	0	0	0	0	0	0	0
Cartilage disruption	2	2	2	2	2	2	2	3	0	0	0	0	0	0	0	0
Hyaline cartilage destruction	2	2	2	2	2	2	2	3	0	0	0	0	0	0	0	0
Subchondral bone destruction	3	3	2	3	2	2	2	3	0	0	0	0	0	0	0	0
Overall assessment: "determined as arthritis"	3	3	3	3	3	3	3	3	0	0	0	0	0	0	0	0

Histopathological changes in the joints from mAb-induced arthritis were described and scored, using semi-quantitative grading of five scores (0, unremarkable, 1, minimal, 2, mild, 3, moderate, 4, marked).

### Apremilast reduces severity of CIA in DBA/1 mice

**Figure 3:**
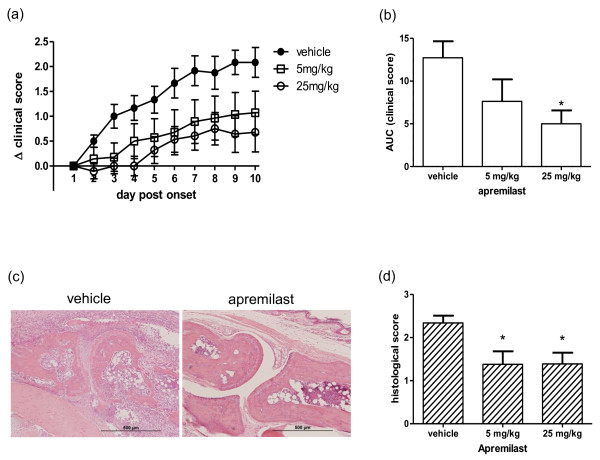
**Apremilast reduces severity of collagen-induced arthritis in DBA/1 mice**. Arthritic, male DBA/1 mice were treated from day one of onset to day 10 post onset of arthritis with a daily, intra-peritoneal dose of apremilast or vehicle, after which the mice were sacrificed and paws were removed for histological analysis. Disease severity was evaluated throughout. Change in clinical score is plotted in **(a)**. Each treatment group comprised of 14 mice, with means and standard errors plotted. **(b) **The statistical significance of the clinical score of apremilast-treated compared with vehicle-treated mice was calculated from area under the curve (AUC) for each mouse over the 10 day treatment period (* *P *< 0.05). Representative images of H&E-stained sections through the proximal interphalangeal joint of an apremilast-treated mouse and a vehicle-treated mouse are shown **(c)**. Scale bars are 500 μm. **(d) **All sections were scored for the extent of inflammation and damage, and graded accordingly. (* *P *< 0.05).

The therapeutic effectiveness of apremilast in suppressing experimental arthritis was evaluated further in CIA; a well-established model of murine arthritis involving a single immunisation of male DBA/1 mice with type II bovine collagen in CFA (Figure 3). Fourteen mice per treatment group were given a daily, oral administration of vehicle, or 5 or 25 mg/kg apremilast from day one of onset, to day 10 post onset of arthritis and disease severity was evaluated throughout by means of semi-quantitative clinical score as described in materials and methods. The change in clinical score from day 1 post onset is plotted in Figure 3a. Clinical scores of all mice were comparable (0.5 or 1) when treatment was initiated. Although both doses of apremilast-suppressed arthritis, relative to vehicle, it was clear that the higher dose had a more profound effect. The area under the curve of Δclinical score for each mouse was calculated and statistical significance, relative to vehicle control group, was evaluated (Figure 3b). In accordance with apremilast treatment in mAb-induced arthritis, Δclinical score of mice receiving 25 mg/kg but not 5 mg/kg apremilast was significantly reduced, as compared with those given vehicle only (*P *< 0.01).

In order to assess joint pathology, the affected hind paws of each animal was removed at the end of the experiment and processed for H&E staining. Representative images of H&E-stained sections through the proximal inter-phalangeal joint of a vehicle-treated and an apremilast-treated mouse are shown in Figure 3c. Although the joint of the vehicle-treated mouse is clearly flooded with inflammatory cell infiltrate and displays severe loss of architecture, the joint from the apremilast-treated mouse shows a clear joint space and normal joint architecture with articular cartilage and bone preserved. Sections from proximal inter-phalangeal joints from treated mice were graded according to the parameters detailed in materials and methods. Interestingly, sections from both apremilast treatment groups (5 and 25 mg/kg) displayed significantly reduced joint pathology relative to those from vehicle-control mice (*P *< 0.05, Figure 3d).

### Apremilast lacks adverse effects of rolipram

**Figure 4:**
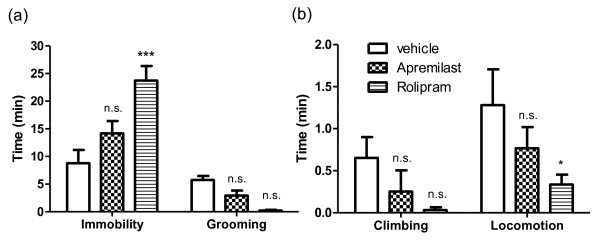
**The effect of 25 mg/kg apremilast or rolipram on spontaneous behaviour was tested on naïve mice using LABORAS automated activity monitor**. The average time spent **(a) **immobile and grooming and **(b) **climbing and during locomotion are plotted. n = 8 per group, * *P *< 0.05, *** *P *< 0.001, relative to vehicle treatment. Mice were monitored for 30 minutes, one hour after receiving treatment. n.s., not significant.

It is well documented that PDE4 inhibitors, including rolipram, can induce a variety of side effects ranging from nausea, vomiting and diarrhoea, to more serious conditions such as vasculitis and colitis (reviewed in [15]). In mice, the main effect we observed following treatment with rolipram is lethargy. To determine whether apremilast triggers similar side effects, treated mice were monitored using an automated activity monitor (LABORAS) to assess multiple activities including immobility, grooming (Figure 4a), climbing and locomotion (Figure 4b) [27,28,41]. Strikingly, mice treated with 25 mg/kg apremilast (i.p.) did not show any significant behavioural changes in any of the parameters tested. As predicted, mice treated with rolipram (i.p.) exhibited significantly increased immobility (*P *< 0.001), reduced grooming (although not statistically significant) and reduced locomotion (*P *< 0.05); factors associated with lethargy (n = 8 per group). Means and standard errors are plotted. Notably, mice treated with either apremilast or rolipram did not experience diarrhoea or significant weight loss. Thus, we conclude that, unlike rolipram, apremilast does not influence spontaneous behaviour and lethargy at a dose that ameliorates CIA and suppresses inflammation.

### Apremilast inhibits antigen-specific T cell cytokine production and proliferation

**Figure 5:**
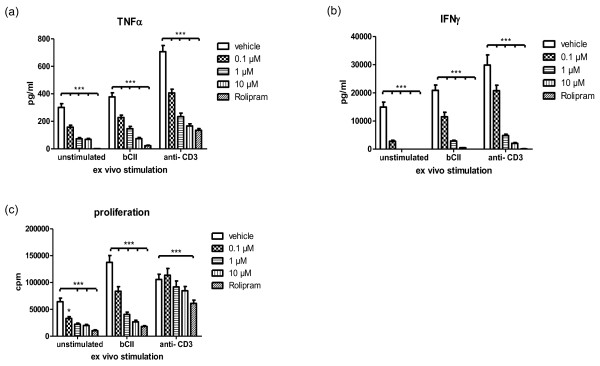
**Apremilast inhibits antigen specific T cell cytokine production and proliferation in lymph node cultures**. Male DBA/1 mice were immunised with bCII in CFA and 14 days later, inguinal lymph nodes were removed and cells were dissociated. Lymph node cultures, unstimulated, or stimulated with either bCII or anti-CD3 mAb, were cultured in the presence of increasing concentrations of apremilast or 100 μM rolipram for 48 hours at which time supernatants were removed and analysed for **(a) **TNFα, and **(b) **interferon (IFN)γ production by ELISA. **(c) **Cells were then pulsed with ^3^H- thymidine for a further 20 hours to assess cell proliferation. Statistical significance between treatment and control groups was calculated by two-way analysis of variance with Bonferroni multiple comparison test. (* *P *< 0.05, *** *P *< 0.001).

Lymph node cultures from male DBA/1 mice immunised with bCII in CFA were unstimulated, or stimulated with bCII or anti-CD3 mAb, in the presence of increasing concentrations of apremilast or 100 μM rolipram, (used as positive control only), for three days. After 48 hours, supernatants were collected for cytokine analysis, before cells were pulsed with tritiated thymidine to measure proliferation (Figure 5). All concentrations of apremilast and rolipram significantly inhibited unstimulated, antigen-specific, as well as total, T cell production of TNFα (Figure 5a) and IFNγ (Figure 5b; *P *< 0.001). Furthermore, antigen-specific T cell proliferation was significantly inhibited in a dose-dependent manner, while only rolipram inhibited total T cell proliferation, as observed in anti-CD3 mAb-stimulated lymph node cultures (Figure 5c). No cytotoxic effects of apremilast were observed in lymph node cultures when routinely assayed by MTT (data not shown).

## Conclusions

Apremilast is a novel, orally available PDE4 inhibitor that inhibits spontaneous TNFα production from human rheumatoid synovial membrane cultures, *ex-vivo*, with similar efficacy to rolipram. In addition, apremilast effectively reduced the severity of both mAb-induced arthritis and CIA in BALB/c and DBA/1 mice, respectively, over 10 days post onset, without any evident side effects, often associated with classical PDE4 inhibitors such as rolipram. Taken together, our results show that apremilast has therapeutic potential for the treatment of RA and other chronic inflammatory conditions where TNFα plays a major pathological role.

## Abbreviations

ANOVA: analysis of variance; cAMP: cyclic adenosine monophosphate; CFA: complete Freund's adjuvant; CIA: collagen-induced arthritis; COPD: chronic obstructive pulmonary disease; DMSO: dimethyl sulphoxide; ELISA: enzyme-linked immunosorbent assay; FCS: fetal calf serum; H&E: haematoxylin and eosin; IC: inhibitory concentration; IFNγ: interferon gamma; IL: interleukin; i.p.: intraperitoneal; LABORAS: The Laboratory Animal Behaviour Observation: Registration and Analysis System; LPS: lipopolysaccharide; mAb: monoclonal antibodies; MTT: 3-(4,5-Dimethylthiazol-2-yl)-2,5-diphenyltetrazolium bromide; PBMC: peripheral blood mononuclear cells; PDE4: phosphodiesterase type 4; RA: rheumatoid arthritis; TNFα: tumour necrosis factor alpha.

## Competing interests

Peter Schafer is an employee of Celgene Corporation and holds patents on apremilast. Marc Feldmann was a consultant of Celgene Corporation and obtained a research grant from Celgene Corporation to fund this study. All other authors have no competing interests.

## Authors' contributions

FMcC drafted the manuscript and analysed and interpreted the data. AP acquired the data from LPS-stimulated monocytes and RA synovial cells, and contributed to drafting of the manuscript. MA carried out CIA experiments. DP and JI conducted behavioural studies using LABORAS. PS provided apremilast and participated in the design of the study. MF participated in the conception and design of the study. RW and FB participated in the conception and design of the study, contributed to analysis and interpretation of data and assisted in drafting the manuscript. All authors approved the final manuscript.

## Supplementary Material

Additional file 1 **Supplementary figure S1. Apremilast has no effect on cell viability in human cells**. (a) Human monocytes and (b) rheumatoid arthritis (RA) synovial membrane cells were cultured with increasing concentrations of apremilast or rolipram as in Figure 1. After supernatants were collected for cytokine analysis, 3-(4,5-Dimethylthiazol-2-yl)-2,5-diphenyltetrazolium bromide (MTT) was added at a final concentration of 0.5 ng/ml for six hours. A 100 μl sample of 10% SDS in 0.01 M HCl was then added overnight before the plate was read on a spectrophotometer at 620 nm. None of the culture conditions assayed altered cell viability relative to cells alone. Click here for file
