# Supplementary figures and images for: Apremilast, a novel PDE4 inhibitor, inhibits spontaneous production of tumour necrosis factor-alpha from human rheumatoid synovial cells and ameliorates experimental arthritis
Source: Arthritis Res Ther. 2010 Jun 2;12(3):R107. doi: 10.1186/ar3041 (PMC2911898; doi:10.1186/ar3041)

Supplementary figure 1

(a)

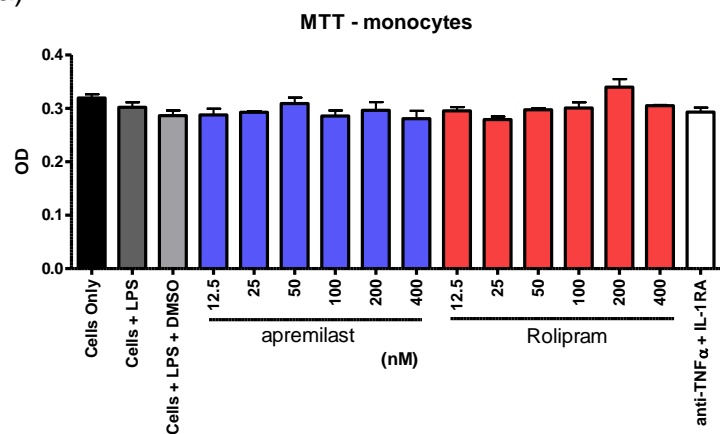

(b)

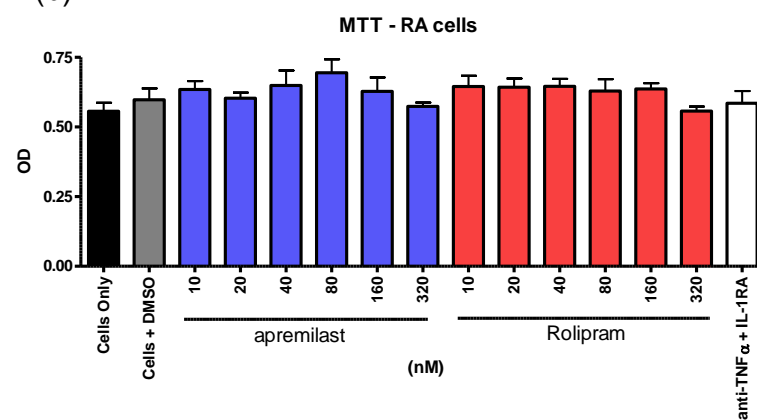

Supplement: Additional file 1 — Supplementary figure S1. Apremilast has no effect on cell viability in human cells. (a) Human monocytes and (b) rheumatoid arthritis (RA) synovial membrane cells were cultured with increasing concentrations of apremilast or rolipram as in Figure 1. After supernatants were collected for cytokine analysis, 3-(4,5-Dimethylthiazol-2-yl)-2,5-diphenyltetrazolium bromide (MTT) was added at a final concentration of 0.5 ng/ml for six hours. A 100 μl sample of 10% SDS in 0.01 M HCl was then added overnight before the plate was read on a spectrophotometer at 620 nm. None of the culture conditions assayed altered cell viability relative to cells alone. [file ar3041-S1.PDF]
